# Farmers’ trust in government and participation intention toward rural tourism through TAM: The moderation effect of perceived risk

**DOI:** 10.3389/fpsyg.2022.1023280

**Published:** 2022-11-03

**Authors:** Xia Yu

**Affiliations:** ^1^College of Tourism and Leisure Management, Fujian Business University, Fuzhou, Fujian, China; ^2^College of Economics and Management, Fujian Agriculture and Forestry University, Fuzhou, Fujian, China

**Keywords:** rural tourism, perceived risk, government trust, perceived usefulness, participation intention

## Abstract

At present, there are almost 700 million rural population in China, and the farm and farmers in China are highly associated with the steadiness and development of the country and even the world. Farmers are the main subjects in rural development and play a vital role in the reception, management, and benefit distribution in rural tourism activities during the development of rural tourism. Farmers’ perception and participation intention in rural tourism development are directly related to the sustainable development of rural tourism and the realization of rural revitalization goals. The decision-making process of participation in the rural tourism development fits the application conditions of the technology acceptance model (TAM). Therefore, in order to explore the influencing factors of farmers’ decision-making process in participating in the rural tourism, this study employs the technology acceptance model to predict and judge individual farmers’ willingness and behavior to participate in the rural tourism. The government trust and perceived risk in the real problem of low participation of farmers in the rural tourism development are considered. Then, an extended technology acceptance model was established by taking 409 farm households as research samples. The influence of government trust on farmers’ participation intention in the rural tourism was empirically analyzed based on the PLS-SEM model. The results show that farmers’ perceived usefulness and perceived ease of use in rural tourism affect their participation intention. Farmers’ judgments on whether governments can assume public responsibilities and achieve public interests through rural tourism development affects their trust in government, while the government trust can positively affect farmers’ participation intention *via* perceived usefulness and perceived ease of use. This indicates that government trust is an important antecedent variable influencing farmers’ participation in the rural tourism. The perceived risk affects farmers’ perceived usefulness and participation intention in the rural tourism, and plays a moderating role in the relationship between government trust and perceived usefulness. Finally, this study recommends to highlight the utility and convenience of rural tourism participation during the promotion of farmers’ participation in rural tourism development, enhance the ability of farmers to participate in rural tourism development, and choose multiple channels to increase government trust to reduce farmers’ risk concerns.

## Introduction

The report of the 19th People’s Congress of the Communist Party of China puts forward the general requirement of implementing the rural revitalization strategy for the first time. This strategy emphasizes the need to enhance the comprehensive competitiveness of agriculture and promote the integration of the three rural industries. It also encourages farmers to be employed and start their businesses to achieve income growth, thereby achieving the modernization in agriculture and rural areas. Many studies (Gao and Wu, 2017; Liu et al., 2020; Chen et al., 2022) have proved that rural tourism plays a remarkable role in developing the agricultural economy, improving the rural appearance and farmers’ income, providing employments, and other functions, which is an effective means of poverty reduction. The income of rural tourism mainly comes from catering, lodging, transporting, visiting, shopping and entertainment in the process of rural tourism consumption, while the total tourist arrivals can be calculated by multiplying the number of trips and the number of rural tourists. The income of rural tourism and the total tourist arrivals are two significant indicators of rural tourism economy. In 2021, the income of rural tourism in China achieved 630 billion yuan, increasing by 50% over the same period in the previous year; the total tourist arrivals of rural tourism reached 1.622 billion, increasing by 14.55% compared to the same period of last year. Rural tourism has become an important direction to the high-quality tourism development which pursue development quality instead of quantity under the epidemic (Chi and Han, 2020) and a new growth point for rural economic development in the new era. Rural tourism, with its green and ecological characteristics, has gradually developed into the landing place and main position for rural revitalization. The “Guidance of the State Council on Promoting the Revitalization of Rural Industries” issued by China in June 2019 explicitly puts forward the requirement of increasing farmers’ participation and establishing a mechanism for promoting farmers’ participation in the development of industries. The 2020 China’s Central Document No. 1 also clearly points out the need to establish a sound mechanism for farmers to share the value-added returns of the industrial chain and integrate small farmers into the agricultural industrial chain.

Meanwhile, the people-centered development concept was proposed based on the requirements of new era. During the development of rural tourism, farmers are the main players in the activities of rural tourism reception, management and benefit distribution. They are also the core subjects of rural tourism development both from the theoretical and practical levels. In recent years, As the problem of agriculture, country and rural residents in China is becoming more and more important, problem of farmers has received much attention. Therefore, raising the participation level of farm households and personalizing the precise policy-making from the top-level design level have been the unified understanding and the necessary way for rural revitalization in China. Farm households are the basic production, living, and distribution units of rural communities in China at the micro level. Agrotourism, a usual and predominant kind of rural tourism, has been proven an excellent way to raise the income of farm households, enhance livelihoods, lessen poverty, and regenerate rural communities (Su, 2011; Park et al., 2019). Therefore, in order to consolidate the results of poverty eradication and achieve common prosperity, guiding and helping rural households to participate in the rural tourism is one of the important issues in China’s current development of rural tourism. Among them, farmers’ participation intention in the rural tourism and its influence variables are important links to stimulate the vitality and potential of the farmer subjects. How to improve farmers’ participation intention in the rural tourism development and thus promote the sustainable and stable development of rural tourism is also a hot topic in rural tourism research (Lu et al., 2017; Zhang and Niu, 2022).

Scholars have conducted a great deal research on the rural tourism participation in recent years, including the importance and connotation of participation (Wang et al., 2022), residents’ perceptions on the effects of tourism poverty alleviation, the practical framework of rural poor communities’ participation in tourism development and its planning (Ding, 2006), the mechanism of participatory tourism poverty alleviation (Yang and Ba, 2012), and the improvement of the legal mechanism of participating in tourism poverty alleviation (Cao et al., 2014). These results have greatly enriched the research in the field of rural tourism participation in China, but there are few in-depth and detailed empirical studies. Farmers’ participation in rural tourism is influenced by a variety of complicated factors, as well as their interactions. Thus, to study the problem of farmers’ participation which is treated as resultant variable, we have to examine these factors which act as antecedent variables and their formation mechanisms. Scholars have mainly used the Probit, Logistic, and Logit models to perform in-depth quantitative analysis on “the factors that influence farmers’ participation intention in tourism and their behaviors” (Lu et al., 2017) and “the impact of rural tourism development on farmers’ livelihoods” (He et al., 2014; Sun et al., 2020). Since traditional regression models cannot effectively deal with variables that cannot be directly observed, scholars have recently attempted to apply structural equation models to examine the factors influencing farmers’ participation and achieved some progress (Gao, 2015). Some scholars have tried to apply theoretical models such as the theory of planned behavior and the theory of perceived value to construct an analytical framework for farmers’ decision-making process in tourism participation (Radic et al., 2022). However, there are few theoretical frameworks in related studies, and the integrated application of such theories is relatively rare. The reason maybe that rural tourism participation is a systematic project, and the decision-making process of farmers is influenced by various factors, such as participation benefits, participation ability, and participation risks. In the existing studies, these important factors that affect farmers’ participation intention are not fully considered in the analytical framework. Besides, government trust has a crucial influence on farmers’ rural tourism participation behavior due to China’s national conditions and traditional culture. However, most of the existing studies involving government trust-related topics take government policy as a variable. They are mostly related to the variable of government policy, and only stay at the level of explaining the policy content (Sun et al., 2020). Few studies take the government trust and tourism participation as two parallel front-end variables, and the relationship between these two variables has not been discussed in depth. Meanwhile, there is a lack of in-depth discussion on the influencing mechanism of rural tourism participation of farmers from the perspective of government trust.

Therefore, the study examines the influence of government trust on farmers’ participation intention in rural tourism and its formation mechanisms. Based on the existing studies and realistic situations, an extended technology acceptance model was proposed by incorporating government trust variables based on the traditional TAM model, in which the perceived risk was used as a moderator. Using this model, the influence of government trust on farmers’ rural tourism participation and its transmission mechanism was explored. Perceived risk, as individuals’ subjective beliefs or assessment concerning an uncertain situation (Han et al., 2021), is applied to anlyze the moderation effect. This paper aims to figure out the following questions: First, whether the extended TAM model can be applied as the research model of this paper to study farmers’ participation intention in the rural tourism; Second, how the mechanism of government trust as an antecedent variable of the extended TAM model on farmers’ participation intention in the rural tourism is formed; Third, how the perceived risk moderates in the relationship between the influence of government trust on perceived usefulness and perceived ease of use and the participation intention.

The main contributions of this paper are as follows. First, most of the existing studies on farmers’ rural tourism participation behavior use the theory of planned behavior, and few scholars have used the TAM model to predict and judge individual farmers’ willingness and behavior to participate in the rural tourism. As the application of TAM models continues to expand, the trend of interdisciplinary and introduction of new theories and variables has been highlighted (Lv, 2022). The decision-making process of farmers’ behavioral intentions also fits well with the conditions and decision-making process in the application of the TAM model. Therefore, compared with previous studies that focus on the impact of technology functionality on individual acceptance behavior, this study first makes a breakthrough and establishes an extended TAM model to measure the “perceived usefulness” and “perceived ease of use” of farmers’ participation in the rural tourism and analyze their behavioral intentions. This can enrich the study of the TAM model. Second, the research on government trust in tourism-related fields is rare, and the rare research that considers government trust and participation behavior as parallel antecedent variables does not provide an in-depth analysis of the relationships and paths between them (Wang and Lu, 2014). Realistically, Chinese government has a pivotal role in the rural tourism development, and the government trust affects farmers’ perceptions of tourism and participation intention. Therefore, this study combines government trust as a predictor variable into the model to investigate the path and effect of government trust on farmers’ participation intention in the rural tourism. Third, the impact of risk factors on farmers’ government trust and rural tourism participation behavior is also considered, aiming to analyze whether the perceived risk can play a moderating role in the relationship between the influence of government trust on perceived usefulness, perceived ease of use, and participation intention. Based on this, the microscopic mechanism of the effect of perceived risk on farm households’ rural tourism participation behavior is revealed.

## Literature review and theoretical basis

### Technology acceptance model

The Technology Acceptance Model (TAM) is a classical model proposed to study the individual acceptance behavior of new technologies and products based on the theory of planned behavior (Yu et al., 2005). The core of the TAM model is that the use of new technologies by individual “rational economic man” is determined by behavioral intentions and influenced by two core factors, i.e., perceived usefulness and perceived ease of use. Thus, the TAM model can be considered an open model allowing to add, extend, and improve from different perspectives (Lee, 2009; Liu and Liu, 2015; Luo and Zhu, 2015). The existing studies have integrated internal and external variables such as trust, risk, return, policy, and perceived behavioral control conditions to validate and improve the explanatory power of the model, and applied this model to other areas such as technology adoption, consumer willingness to purchase behavior, and farmer participation (Davis, 1985; Fang and Li, 2021; Wang et al., 2021).

As a new field, technology, and production method, rural tourism is influenced by individual and family factors, perceived interests, economic and social capital, government support and policy systems, and risks in the development process. This usually leads to differences in farmers’ participation intention and levels of participation. Farmers will carefully consider the benefits and losses in the decision-making process for rural tourism participation, objectively assess the capabilities and resources, and then form behavioral preferences. The assumption that farmers are rational economic men and the decision-making process of behavioral intentions agree well with the TAM model. Therefore, the TAM model can be applied to measure the “perceived usefulness” of farmers in participating in the rural tourism and compare the “perceived ease of use” of farmers to analyze their behavioral intentions. To date, there have few studies in exploring the relationship between farm households’ rural tourism participation and behavioral intentions using the TAM model, and the relationship between them needs to be further clarified.

### Government trust

Rural tourism development involves coordination and cooperation among multiple interest subjects. Different interest subjects constitute an intricate network of social relations. As the core stakeholders in rural tourism development, mutual trust between the government and residents is crucial for the sustainable tourism development. The government regulates the rural tourism development through high-level promotion, industrial planning, and institutional supply. Farmers assess the trustworthiness of governments based on the results of government policies and behaviors, such as political and economic performance, cost inputs, and government services in tourism development (Nunkoo and Gursoy, 2017). Trust, as a complex social and psychological phenomenon, is based on attitudes and confidence toward others from a cognitive and psycho-emotional perspective (Nguyne and Rose, 2009).

Residents in rural tourism development form subjective perceptions and judgments on whether governments can assume public responsibilities and achieve public interests and goals during the rural tourism development. Such perceptions and judgments greatly influence residents’ attitudes, judgments, and the acceptance of tourism development (Nunkoo and Gursoy, 2016). Government trust has been explored in many areas such as the willingness to transfer agricultural land, urban residents’ participation in environmental governance, and public risk perceptions in epidemics (Pu and Yuan, 2018; Wu, 2021; Zhu et al., 2022). The research results have gradually attracted the attention of the tourism community (Jia et al., 2021). Currently, the research on government trust in tourism-related fields is relatively rare and needs to be further enriched and improved.

### Perceived risk

The game of interests between various interest subjects in the process of rural tourism development may lead to the marginalization of farmers. Farmers have to undertake the result of tourism development, which comes with unpredictability. Due to the peasant mindset of “satisfaction with small achievements” brought by the traditional Chinese farming civilization, farmers’ spirit of risk-taking, sense of innovation, and ability to recognize and accept new things are relatively lacking. They tend to be risk averse when faced with uncertain outcomes. The unpredictability of the consequences for farmers’ participation behavior in the rural tourism is the risk, such as the concerns about participation costs, participation benefits, participation capacity, and changes in survival methods and means.

Depending on the national context and farmers’ perceptions in Chinese governments, the benefits, costs, and improvements in living standards brought by tourism development are considered as the effectiveness of government policy implementation. This further affects their interaction and levels of trust. Concerns about risk can therefore invariably affect farmers’ propensity to trust the government and their participation intention in the rural tourism (Sun et al., 2020). Existing studies generally agree on the impact of risk perception on government trust. For example, Egea and González (2011) studied the physician adoption behavior toward electronic medical records and found that the perceived risk can affect the government trust (Egea and González, 2011). However, there is no consensus among the existing studies regarding the direction of the role between government trust and risk perception. Most studies show a negative correlation between government trust and public risk perception, that is, the higher level the trust in government, the higher level the risk perception of people (Victoria et al., 2009; Han et al., 2017; Fridman et al., 2020). Zhu et al. (2022) explained the mechanism of the effect of government trust on risk perception with the help of squeeze effect and compliance effect. The results showed that the government trust can negatively and slightly affect the public risk perception, and the direction of government trust on the public risk perception was consistent with the direction required by government risk communication goals.

### Model construction and research hypotheses

**Figure 1:**
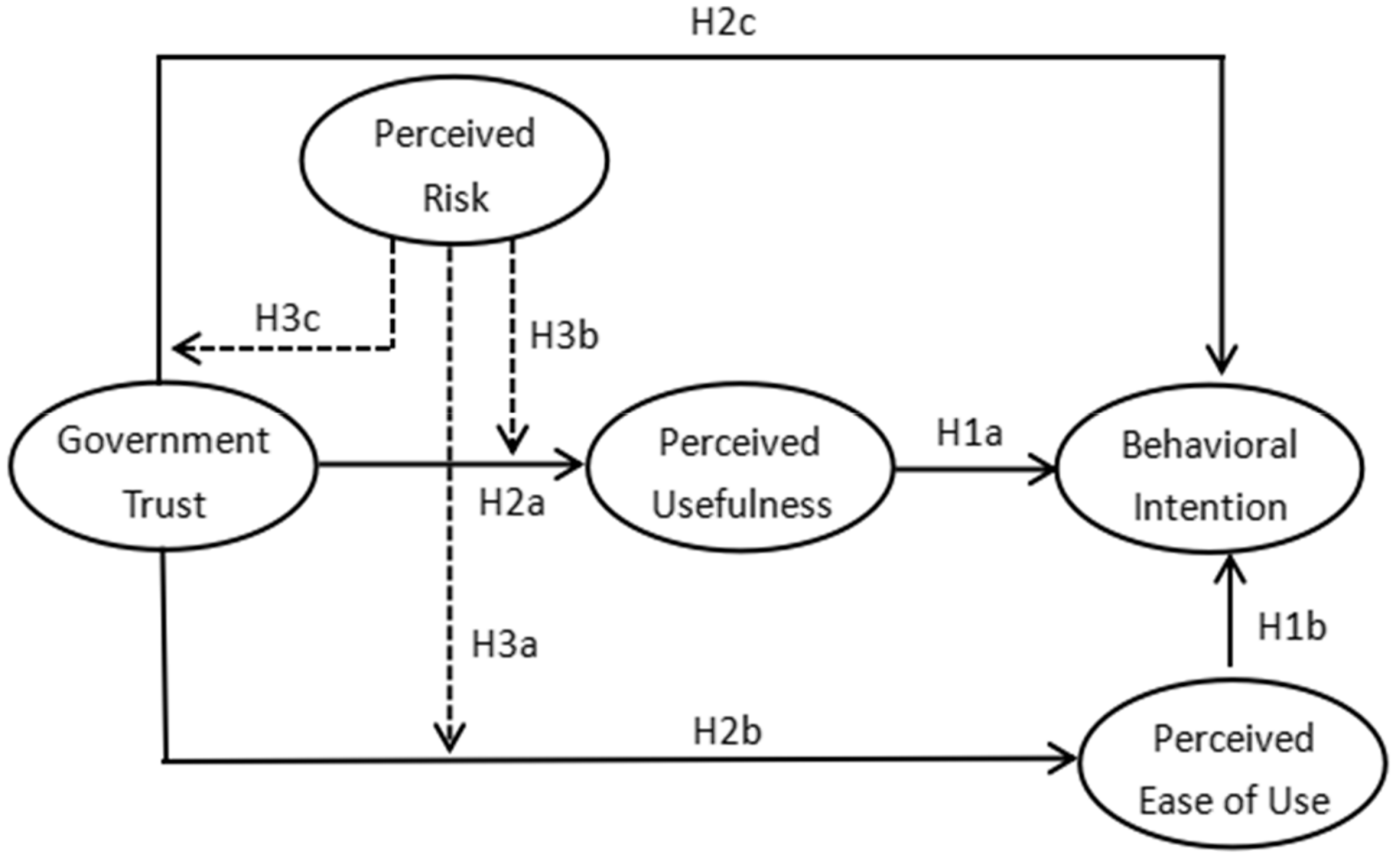
Conceptual model.

The traditional TAM model, government trust theory, and perceived risk theory were combined to construct an extended TAM model for farmers’ participation intention behavior in the rural tourism development (Figure 1). First, two main variables of the TAM model, i.e., perceived usefulness and perceived ease of use, were selected as latent variables to measure farmers’ participation intention. Then, government trust was introduced as an antecedent variable of the TAM model to explore the mechanism of the effects of government trust on the rural tourism participation. Finally, the perceived risk was incorporated as a moderating variable of government trust to analyze the moderating effect of the perceived risk on the relationship between government trust and farmers’ participation intention in the rural tourism, which enhances the explanatory power of the model.

### Perceived usefulness, perceived ease of use, and farmers’ participation intention

Rural tourism development can boost local economic development, create employment opportunities, and increase farmers’ income. It can also cause a series of negative impacts, such as price increases, traffic congestion, security problems, and environmental damages (Nunkoo and Gursoy, 2012). Farmers’ supporting and participation behavior in rural tourism development can be determined after perceiving, judging, and weighing the impacts of tourism (Ward and Berno, 2011; Latkova and Vogt, 2012). Perceived usefulness is the benefits that farmers subjectively perceive to be brought by participating in the rural tourism development, which is the key to their participation (Du and Jing, 2019). Farmers’ perceived usefulness in the rural tourism development has a positive effect on their participation intention (Nunkoo and Ramkissoon, 2012). If farmers perceive the positive impacts that would be brought by rural tourism development (e.g., local economic development, higher household income, more employment opportunities), they will show a positive participation intention and promote the participation behavior. Conversely, their participation intention will diminish. Most of the current literature finds that users show a positive willingness toward a technology with a more useful use that can be perceived. Therefore, we propose the following research hypothesis.

*H*1a: Farmers’ perceived usefulness of rural tourism has a positive effect on their participation intention.

Limited by factors such as literacy, age, access to capital, and the ability to accept new things, farmers are also concerned about the threshold, difficulty, and risk of participation in the rural tourism. The perceived ease of use refers to the degree of difficulty and effort required to participate in the rural tourism as subjectively perceived by farmers. Studies have shown that the perceived ease of use contributes to personal preferences, reduces anxiety about new things, and thus influences individual’s behavioral intentions (Davis et al., 1989; Brown et al., 2004). When farmers perceive that the threshold and difficulty of participating in the rural tourism are low or that they can participate in rural tourism activities with less effort (e.g., elemental information related to the participation behavior in the rural tourism such as fields, mountains, forests, and houses are easily available, and new technologies and methods are easier to master), they tend to show a more positive intention to participate in. The likelihood of their participation in the rural tourism is then increased. Most studies agree that the perceived ease of use has a positive effect on individual’s behavioral intentions, but there are also inconsistent findings (Li et al., 2010). Therefore, we propose the following hypothesis.

*H1b*: Farmers’ perceived ease of use of rural tourism has a positive effect on their participation intention.

### Government trust, perceived usefulness, perceived ease of use, and farmers’ participation intention

Rural tourism development is usually fraught with a variety of risks from natural and social, economic and market sources, and uncertainties in the expected outcomes. Trust among stakeholders such as government, enterprises, and farmers, especially farmers’ trust in government, is an important prerequisite for the development of rural tourism. The level of farmers’ trust in the government affects their perceptions of expected outcomes and participation intention in the rural tourism (Gursoy et al., 2017). The higher the level of trust in the government, the more likelihood of farmers to approve local government’s decision on the development of rural tourism. They also have more trust in government’s efforts to pursue local economic development and improve people’s lives, starting from the perspective of residents and committing to maximizing benefits and minimizing difficulties. Thus, at high levels of government trust, farmers show more optimism about the expected benefits and costs associated with rural tourism development (Nunkoo and Gursoy, 2012), and the perceived usefulness of rural tourism development will be more significant. To be specific, the higher the level of government trust, the higher the expectation of farmers to receive various types of training, guidance, and assistance, the more significant the perceived ease of use of tourism development, and the stronger the willingness of farmers to participate in. Conversely, farmers with low trust in the government show more concerns about the possible negative effects of tourism. The possible risks and difficulties faced during the rural tourism development will be magnified, thus reducing their participation intention. Many studies have also verified the effect of government trust on farmers’ behavioral intentions. For example, Lu et al. (2017) empirically analyzed the impact of government trust of Tibetan farmers and herdsmen on their subjective norms and behavioral attitudes. Pu and Yuan (2018) analyzed the relationship between farmers’ farmland transfer decision-making behavior and government trust. Nunkoo and Gursoy (2012) found that the trust level of residents in government not only directly influenced their tourism participation but also had a positive impact on the participation intention and behavior based on the perception of rural tourism. Therefore, it can be hypothesized that on one hand, the government trust can directly affect farmers’ participation intention in the rural tourism, and on the other hand, it has an indirect influence through the mediating effects of perceived usefulness and perceived ease of use. Therefore, the following research hypotheses are proposed.

*H2a*: Government trust has a positive effect on the perceived usefulness of farmers’ participation in the rural tourism.

*H2b*: Government trust has a positive effect on the perceived ease of use of farmers’ participation in the rural tourism.

*H2c*: Government trust has a positive effect on farmers’ participation intention in the rural tourism.

### Moderating role of perceived risk

The perceived risk theory was first introduced into the field of consumer behavior by Bauer, who argued that the uncertainty in consumption outcomes poses decision risks (Bauer, 1960). Pidgeon empirically tested that perceived risk is a direct determinant of individual attitudes and decision behavior (Pidgeon et al., 1992). Taylor analyzed the patterns of individual uncertainty-reducing behavior in risk-perception situations (Taylor, 1974). These studies have shown that perceived risk is the basis of individual behavioral predictions and behavioral attitudes, which influences individual’s behavioral attitudes and decision-making behavior. It is an external variable that is generally agreed upon by scholars and has been frequently utilized by technology acceptance models. Rural tourism development has investment and revenue risks and technical thresholds. The effectiveness of development is also limited by the literacy level of farmers and market consumption capacity and implies the risk of lifestyle change. In case that the rural tourism development cannot meet the rational choice to improve the quality of life, or that the rural tourism development may lead to the consequences cannot be accurately foreseen, farmers will perceive the risks such as capital loss, labor and time consumption, life disruption, and psychological unhappiness, which in turn affect the perceived usefulness of rural tourism and farmers’ participation intention. Similarly, if a high “technical threshold” or “participation threshold” that requires extensive knowledge and experience, skilled skills, or adequate financial and factor security is perceived during the rural tourism development, farmers will take the lack of experience, competence, financial risk, and the opportunity cost of factor inputs as risks. Therefore, the perceived ease of use of rural tourism will be reduced, and farmers tend to maintain the status quo and reduce the probability of participation (Lv and Chen, 2010; Barham et al., 2014).

Influenced by China’s national conditions and traditional cultural values, most of farmers lack the knowledge and ability to identify and deal with risks in a preventive manner. They are more inclined to rely on the guidance of government agencies. Thus, government trust is an important way to effectively reduce individual’s risk judgment based on their own experience (Sun et al., 2020). Usually, when the level of government trust is high, farmers are more optimistic about the perceived benefits and costs associated with rural tourism development, and tend to be less wary of the threshold and difficulty of participation, which in turn reduces their perceived risk in the rural tourism development; when the level of government trust is low, farmers rely more on intuition and experience to make decisions (Payne et al., 1996), and their certainty against risk is reduced (Kinzler and Speike, 2007). Currently, many studies have concluded that farm households’ trust in the government has a positive guidance on the participation intention. However, some studies showed that despite of a low level of government trust, residents still actively support and respond to government policies and actions (Han et al., 2017; Ouyang et al., 2017). Since government trust is an important antecedent variable in the study of tourism perceptions and participation intention in the rural tourism (Nunkoo and Gursoy, 2016), it is inferred that in the path of government trust and farm households’ participation intention, there maybe some variables that moderate between the government trust and the tourism perceptions and participation intentions (Draper et al., 2011). In the process of rural tourism development, if farmers concern more about the participation behavior, i.e., higher risks are perceived in the participation behavior, the risk-averse farmers will become more cautious in the rural tourism participation decision-making. Therefore, even though the higher level of government trust can enhance farmers’ perceived usefulness and perceived ease of use of rural tourism and promote their participation intention, the effect of government trust of farmers on their tourism perceptions and participation intention may be moderated by the perceived risk in high-risk situations. Moreover, farmers’ perceived risk may moderate the direct relationship between government trust and rural tourism participation intentions, and may also indirectly affect rural tourism participation intentions by moderating the direct relationship between government trust and tourism perceptions. Accordingly, the following hypotheses are proposed.

*H3a*: Perceived risk plays a moderating role between government trust and perceived usefulness in farmers’ rural tourism participation behavior.

*H3b*: Perceived risk plays a moderating role between government trust and perceived ease of use in farmers’ rural tourism participation behavior.

*H3c*: Perceived risk plays a moderating role between government trust and participation intention in farmers’ rural tourism participation behavior.

## Research design

### Data collection

Due to the impact of epidemic normalization and prevention, the formal survey was carried out from April to August 2022. Considering the availability of data as well as the rural tourism resource endowment, industry base, and project demonstration, questionnaires were distributed in 18 rural tourism sites at different development stages (i.e., initial period, developing period, and maturity stages) in Fujian, Zhejiang, Anhui, and Guizhou provinces through an online platform and peer-to-peer support to ensure the representativeness of the data and the reliability of the empirical results. The randomly sample method was used, and the respondents were required living in rural areas or there were rural tourism destinations nearby. A total of 445 questionnaires were distributed and 428 questionnaires were collected. After excluding the invalid questionnaires those with inconsistent answers to the screening question and obvious patterns in filling out the questionnaire, 409 valid questionnaires were obtained, with a valid recovery rate of 92%.

Among all the valid samples, there were more males than females (Table 1), with an age span between 19 ~ 67 and an average age of 42.956 years. The average year of education was 10.115, which was roughly comparable to the average year of education of the working-age population in China in 2021 (i.e., 10.9 years), but higher than the number of rural residents nationwide. This maybe relate to the adoption of online research in this part, excluding the farmers without access to the Internet and groups that do not know how to use smartphones from the survey. The average monthly income was about 4,285 yuan, and there were a few extreme cases where the overall income span varied widely. The vast majority (81.7%) had no village cadre experience, the average household size of respondents was 4.469, and 27.4% of households were involved in rural tourism operations.

**Table 1:** Respondent’s profile (*N* = 409).

**Items**	**Definition**	**Mean value**	**Maximum value**	**Minimum value**	**Standard deviation**
Gender	1 = male; 0 = female	0.545	1	0	0.4998
Age	Age of the farmer (years)	42.956	67	19	12.386
Education	Number of years of education in the farm household (years)	10.115	22	0	4.406
Monthly income	Monthly income of the farming household (yuan)	4285.012	32,000	0	3346.04
Experience of village cadres	1 = Has experience as a village cadre; 0 = No experience as a village cadre	0.183	1	0	0.387
Participation in rural tourism business	1 = Someone in the household is involved in rural tourism 0 = No one in the household is involved in rural tourism	0.274	1	0	0.446
Family size	Average family population	4.469	14	2	1.581

Author’s estimations.

### Concept measurement

The five-point Likert scale was used to measure the questionnaire, with 1 representing strongly disagree and 5 representing strongly agree. The questionnaire contains two major parts: demographic characteristics survey and variable measurement. Among them, demographic characteristics include the information of gender, age, years of education, monthly income, household size, and occupation. The variables were measured based on multiple indicators. All measurement items were derived from established scales developed by mainstream scholars at home and abroad, combining with the characteristics and attributes of farm households and rural tourism. A small sample test survey was first conducted in December 2021, and representativeness tests, factor analysis, and reliability tests were performed based on this test. The questionnaire was then further simplified, modified, and improved in terms of questions, language expressions, etc. The questionnaire was finally completed based on the test results and expert validation. The measurement results of indexes and their reference sources are shown in Table 2.

**Table 2:** Respondent’s profile (*N* = 409).

**variables**	**Definitions**	**Items**		**References**
Perceived usefulness (PU)	Farmers perceive the utility of participating in the rural tourism development	Contribution to local economic development	PU1	Davis et al. (1989), Chung et al. (2015)
		Enrichment in rural life	PU2	
		Increasing in family income	PU3	
		Employment supply	PU4	
		Improvement in personal capabilities	PU5	
Perception Ease of use (PEOU)	Farmers perceive the threshold and difficulty of participating in the rural tourism development	Resources and conditions for participation	PEOU1	Davis et al. (1989), Pavlou (2003)
		Financial resources for participation	PEOU2	
		Knowledge and skills for participation	PEOU3	
		Possessing opportunities to participate in if desired	PEOU4	
Perceived risk (PR)	Farmers’ perceptions of uncertainty in the outcome of participation in the rural tourism development	High risk of rural tourism	PR1	San-Maritin et al. (2015), Xu et al. (2011)
		High cost of investment	PR2	
		Uncertainty of outcome	PR3	
		Risk of being trapped	PR4	
Government trust (GT)	Farmers’ perceptions of government organizations assuming public responsibilities and benefits	Trust in the government’s decisions in the tourism development	GT1	Nunkoo and Gursoy (2012)
		Believe that the government will consider the interests of the village	GT2	
		Trust in the rationality of government decisions	GT3	
		Trust that the government will exert the utmost effort for the development of village	GT4	
participation intention (WOA)	The possibility of farmers’ participation in the rural tourism development	Willing to participate directly in the rural tourism activities	WOA1	Ahn et al. (2004)
		Willing to indirectly participate in the rural tourism	WOA2	
		Willing to actively recommend others to participate in the rural tourism	WOA3	

The perceived usefulness is measured by “contribution to local economic development,” “enrichment in rural life,” “increasing in family income,” “employment supply,” and “improvement in personal capabilities.” The perceived ease of use is measured by “resources and conditions,” “financial resources,” and “knowledge and skills” required for participation, as well as the “possessing opportunities to participate in if desired.” The perceived risk includes “high risk of rural tourism,” “high cost of investment,” “uncertainty of outcome,” and “risk of being trapped.” Government trust is based on the “trust in the government decision-making in tourism development,” “trust that the government will consider the interests of village,” “trust in the rationality of government decisions,” and “trust that the government will exert the utmost effort for the development of village.” On that basis, participation intention can be measured according to the four items, including “direct participation,” “indirect participation,” “will recommend to others.”

## Data and empirical analysis results

### Measurement model

SmartPLS was used to test the intrinsic reliability and validity of the data. The Cronbach’s ɑ and Composite Reliability (CR) were used in the reliability test to verify the degree of consistency of the data. As shown in Table 3, the loadings of all factors range from 0.714 to 0.893, which meet the criterion that factor loadings should be equal to or greater than 0.7. The Cronbach’s ɑ coefficients of the five latent variables range from 0.766 to 0.86, which meet the criterion that the Cronbach’s ɑ coefficient should be equal to or greater than 0.7. The CR values are all between 0.846 and 0.905, showing a good internal consistency (0.7–0.9) (Raza et al., 2021). It indicates that the internal consistency reliability and the composite reliability of the model are good. The validity test was measured by the Average Variance Extracted (AVE), and the results showed that the AVE of all indexes on their respective latent variables ranged from 0.579 to 0.735, meeting the requirement of greater than 0.5 (Fornell and Larcker, 1981). This indicates that the indexes in the extended TAM model have an authoritative theoretical support and each index has a good convergent validity.

**Table 3:** Measurement model results.

	**Items**	**Mean value**	**Loadings**	**α**	**CR**	**AVE**	**VIF**
PU	PU1	4.384	0.714	0.818	0.892	0.735	1.686
	PU2	4.156	0.752				1.798
	PU3	3.861	0.845				2.180
	PU4	4.056	0.832				2.293
	PU5	3.941	0.831				2.099
PEOU	PEOU1	3.961	0.738	0.859	0.904	0.702	1.215
	PEOU2	3.413	0.715				1.701
	PEOU3	3.342	0.826				1.919
	PEOU4	3.159	0.761				1.588
PR	PR1	3.372	0.822	0.766	0.846	0.579	1.924
	PR2	3.403	0.893				2.463
	PR3	3.694	0.814				2.064
	PR3	3.274	0.824				1.875
GT	GT1	3.932	0.834	0.860	0.905	0.703	2.107
	GT2	3.973	0.855				2.081
	GT3	3.751	0.848				2.047
	GT4	4.029	0.815				1.799
WOA	WOA1	3.878	0.808	0.856	0.896	0.635	1.518
	WOA2	4.056	0.888				2.268
	WOA3	3.971	0.874				2.184

PU, Perceived usefulness; PR, Perceived risk; PEU, Perceived ease of use; GT, Government trust; BI, Behavioral intention. Diagonal bold text is the root value of AVE.

The discriminant validity was measured using the method of Fornell and Larcker (1981) and the Heterotrait-Monotrait ratio (HTMT). The diagonally bolded data are AVE root values and the other data are Pearson correlation coefficients, as shown in Table 4. From the table, the diagonal root values are all greater than the Pearson correlation coefficients, which meet the requirements for discriminant validity judgment. As shown in Table 5, the maximum value in the HTMT ratio of correlations is 0.587, satisfying the requirements of smaller than 0.85. Therefore, the measurement model has a good discriminant validity.

**Table 4:** Fornell–Larcker criterion.

	**AVE**	**BI**	**GT**	PEU	**PR**	**PU**
**BI**	0.735	**0.857**				
**GT**	0.702	0.495	**0.838**			
**PEU**	0.579	0.496	0.524	**0.761**		
**PR**	0.703	0.115	0.026	−0.047	**0.839**	
**PU**	0.635	0.593	0.498	0.502	0.124	**0.797**

Items with diagonals are the square root of the AVE. The bold data are AVE root values in table.

**Table 5:** Heterotrait–Monotrait ratio (HTMT) criterion.

	**BI**	**GT**	PEU	**PR**	**PU**
**BI**					
**GT**	0.587				
**PEU**	0.586	0.608			
**PR**	0.153	0.066	0.173		
**PU**	0.7	0.569	0.557	0.144	

Distinct validity was measured by Fornell and Larcker (1981) and Heterotrait-Monotrait HTMT ratio. The diagonally bolded data are AVE root values and the other data are Pearson correlation coefficients as shown in Table 4. The diagonal root values in the table are all greater than the Pearson correlation coefficients, which meet the requirements for differentiated validity judgment. As shown in Table 5, the maximum value in the heterotrait-monotrait ratio of correlations (HTMT) is 0.587, which is smaller than the criterion of 0.85. Therefore, the measurement model has good discriminant validity. Besides that, the variance inflation test VIF was performed and the values were shown in Table 3. The entire construct’s VIF were much less than 5 that shows an excellent value (Hair et al., 2014). Hence, there is no multicollinearity problem in the data.

### Structural model

The bootstrap program in SmartPLS 3 was used to conduct bootstrapping sampling 5,000 times. Then, the blindfolding program was used to obtain the path coefficients, relevant significance, and validity assessment of the structural model. The value of R^2^ indicates the explanatory power of the variables for the model. According to Cohen’s (1988) criteria, R^2^ ≈ 0.19 indicates a weak explanatory power, ≈0.33 indicates a moderate explanatory power, and>0.69 indicates a strong explanatory power. In this study, the value of R^2^ is 0.436, indicating a moderate to strong explanatory power of the model. Q^2^ reflects the relevance of model predictions. According to Chin (1998) study, Q^2^ greater than 0 is eligible, ≈0.02 is weakly correlated, ≈0.15 is moderately correlated, and ≈0.35 is strongly correlated. In this study, the value of Q^2^ is 0.314, which indicates a moderate to strong relevance of the model prediction. The PLS algorithm was executed for 300 iterations to obtain the path coefficients, and the test results are shown in Table 6. Moreover, the path of hypothesis passed the test based on the following criteria: T-value greater than 1.96 and *p*-value less than 0.1.

**Table 6:** Hypothesis testing and strength of the model.

**Hypothesis**	**Effect type**	**Regression path**	**Original sample (O)**	**Standard deviation (STDEV)**	***T* statistics (|O/STDEV|)**	***p* values**	**Remarks**
H1a	Direct Effect	PU → BI	0.367	0.056	6.58	0	Supported
H1b	Direct Effect	PEU → BI	0.212	0.065	3.258	0.001	Supported
H2a	Direct Effect	GT → PU	0.498	0.040	12.302	0	Supported
H2b	Direct Effect	GT → PEU	0.525	0.044	11.872	0	Supported
H2c	Direct Effect	GT → BI	0.200	0.075	2.676	0.007	Supported
–	Direct Effect	PR → PU	0.203	0.060	3.359	0.001	–
–	Direct Effect	PR → PEU	−0.072	0.052	1.375	0.169	–
–	Direct Effect	PR → BI	0.153	0.050	3.035	0.002	–

**Figure 2:**
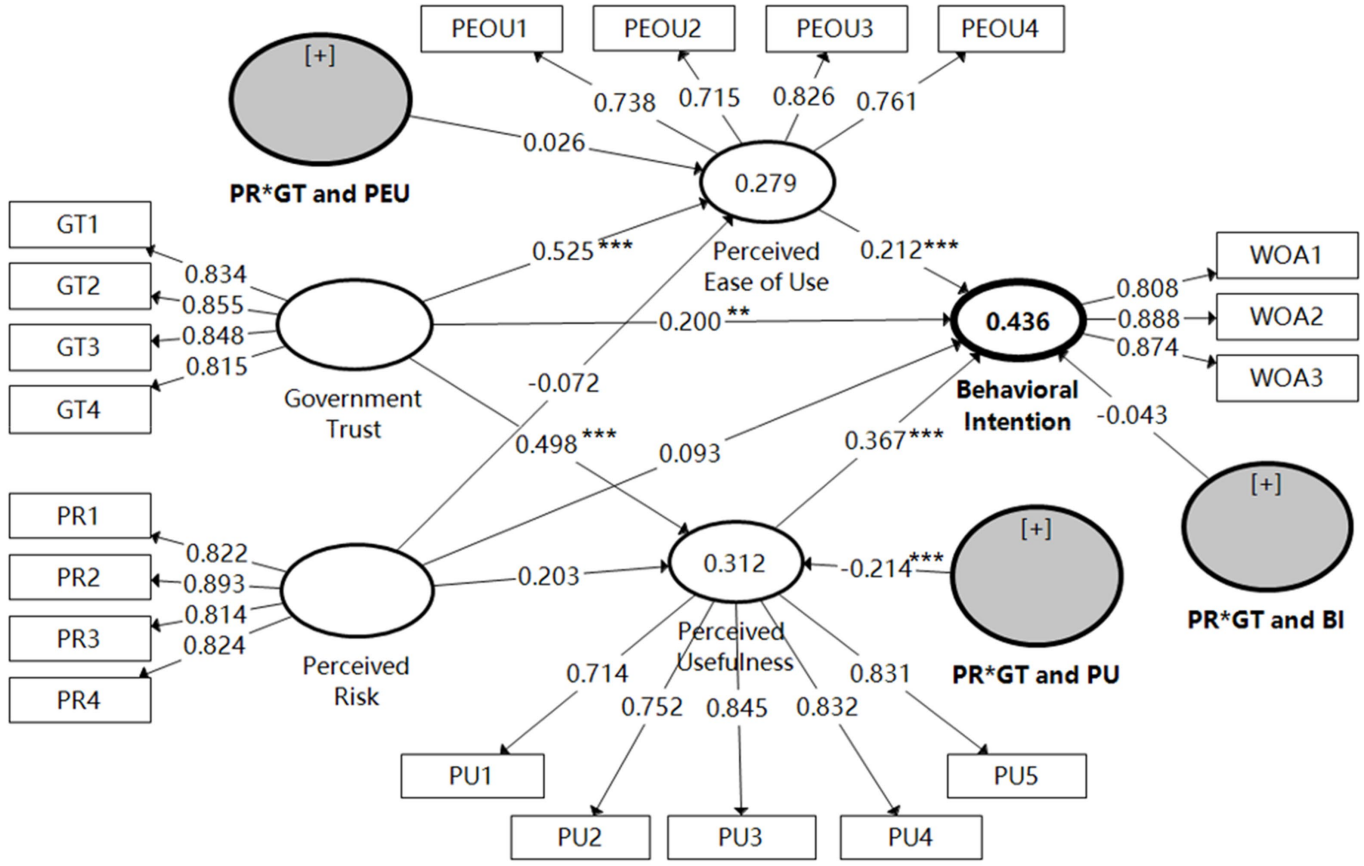
Result of path analysis. **p < 0.01 and ***p < 0.001.

From Table 6 and Figure 2, the perceived usefulness positively influences farmers’ participation intention in the rural tourism (β = 0.367; *T* = 6.58; *p* = 0.000). Therefore, hypothesis H1a was supported (PU → BI). The perceived ease of use has a positive effect on farmers’ participation intention in the rural tourism (β = 0.212; *T* = 3.258; *p* = 0.001), and hypothesis H1b was supported (PEU → BI). Government trust has a positive effect on the perceived usefulness of farm households’ participation in rural tourism development (β = 0.498; *T* = 12.302; p = 0.000). Thus, hypothesis H2a was supported (GT → PU). Government trust positively influences the perceived ease of use of farmers’ participation in rural tourism development (β = 0.525; *T* = 11.872; *p* = 0.000), and hypothesis H2b was supported (GT → PEU). Government trust has a positive effect on farmers’ participation intentions in the rural tourism (β = 0.20; *T* = 2.676; *p* = 0.007), and hypothesis H2c was supported (GT → BI). In addition, the perceived risk positively influences farmers’ perceived usefulness of rural tourism (β = 0.203; *T* = 3.359; p = 0.001) but does not have positive influences on farmers’ perceived ease of use of rural tourism (β = −0.072; *T* = 1.375; *p* = 0.169), mainly due to that the T value was less than the critical value of 1.96 and the p-value was not significantly greater than 0.1. Finally, the perceived risk positively influences farmers’ participation behavior in the rural tourism (β = 0.153; *T* = 3.035; *p* = 0.002).

The above findings suggest that the effect of farmers’ government trust on their participation intention in the rural tourism is mainly mediated by the perceived usefulness and perceived ease of use. In order to clarify whether the facilitation effect of government trust on farmers’ participation intention in the rural tourism is influenced by other factors, it is necessary to further investigate the boundary conditions for the mediating effect. Combined with the above hypothesis, Table 7 lists the test results of the moderating effect of perceived risk in the relationship between government trust and farmers’ participation intention in the rural tourism. First, the perceived risk weakens the role between government trust and perceived usefulness in farmers’ rural tourism participation behavior, playing a negative moderating role (β = −0.214; *T* = 4.772; p = 0.000). The hypothesis H3a was supported. Secondly, the moderating effect of farmers’ perceived risk on the relationship between government trust and perceived ease of use was not significant (β = 0.026; *T* = 0.683; *p* = 0.524), i.e., the T-value was less than the critical value of 1.96, and the value of p was not significantly greater than 0.1. The hypothesis H3b was not supported. Finally, the moderating effect of perceived risk on the relationship between government trust and participation intention was not significant (β = −0.043; *T* = 1.153; *p* = 0.249), i.e., the T-value was less than the critical value of 1.96, and the *p*-value was not significantly greater than 0.1. The hypothesis H3c was not supported. This indicates that the perceived risk significantly inhibits the relationship between government trust and perceived usefulness, but does not moderate in the relationship between government trust and perceived ease of use, or between government trust and participation intentions.

**Table 7:** Moderating effect of perceived risk.

**Hypothesis**	**Regression path**	**Original sample (O)**	**Standard deviation (STDEV)**	***T* statistics (|O/STDEV|)**	***p* values**	**Remarks**
H3a	PR * GT and PU	−0.214	−0.045	4.772	0.000	Supported
H3b	PR * GT and PEU	0.026	0.041	0.683	0.524	Not Supported
H3c	PR * GT and BI	−0.043	0.037	1.153	0.249	Not Supported

## Research conclusion and management implications

### Research conclusion

In the context of China’s rural revitalization, farmers’ participation in rural tourism development has become a concerned area in recent years. In this study, 409 farm households were taken as the research object, and the traditional TAM model was used as the base model for analysis. Taking into account the special characteristics of rural tourism acceptance subjects, acceptance environment, and rural tourism itself, an extended TAM model was proposed by incorporating external variables of government trust. The perceived risk as a moderating variable was also introduced. Using the extended model, empirically tests were performed to examine the mechanisms and paths of the effects of perceived usefulness, perceived ease of use, and government trust on farmers’ participation intention in behavior, and explore the moderating effect of perceived risk. Based on this, the following conclusions were drawn.

First, the extended TAM model is applicable to the study of farmers’ participation intention behavior in the rural tourism. Among the factors directly influencing farmers’ participation intention behavior in the rural tourism, the perceived usefulness has the greatest influence, which is consistent with the assumption of rational economic man. That is, the higher the farmers’ perceived usefulness in promoting local economic development, raising household income, providing employment, and enhancing personal capabilities in the rural tourism development, the stronger the participation intention. Meanwhile, the perceived ease of use is also an important factor influencing farmers’ participation intention behavior in the rural tourism. That is, under the conditions of more adequate the factor conditions (housing, land, forest land, etc.), lower financial threshold, easier available knowledge or skills, and lower difficulties in participation, farmers’ participation intention in the rural tourism development is stronger. The effects of both perceived usefulness and perceived ease of use are greater than 0.2, indicating the fundamental role of the TAM model in the study of farmers’ participation behavior in the rural tourism. This also confirms that farmers as workers in the rural tourism development process are most concerned with usefulness as well as operability, which is in line with the characteristics of farmers. Therefore, in the process of rural tourism development, it is necessary to improve farmers’ cognition level of the rural tourism value, increase the support of elements and funds for farmers, strengthen various types of knowledge and skills training, enhance the participation ability of farmers, and cultivate the endogenous power of rural tourism development.

Second, the improvement of farmers’ trust in the government helps increase farmers’ participation intention in rural tourism development. Farmers’ perceptions of the government are multidimensional and complex. They form different levels of trust in the local government through their intuitive perceptions and judgments on government behavior. There are three paths from the government trust to the behavioral intention, i.e., the direct role of government trust in the behavioral intention (path coefficient of 0.2), the role mediated by perceived usefulness (path coefficient of 0.498), and the role mediated by perceived ease of use (path coefficient of 0.525). This indicates that government trust is mainly mediated by perceived usefulness and perceived ease of use to influence farmers’ behavioral intentions. The more farmers’ trust in government decisions in the rural tourism development, the more likelihood they agree with the government’s efforts for rural tourism development and the government’s protection of farmers’ interests, and the stronger the farmers’ perceptions of expected gain and ease of use. Then, they will show more optimism on the prospect of rural tourism development and stronger participation intention. This finding is consistent with the studies of Nunkoo (2015) and Ouyang et al. (2017), which verified that government trust is an important force that cannot be ignored in influencing rural tourism participation in Chinese rural environment. In addition, it also shows that the outcome of rural tourism development is related to the vital interests of farm households. Ensuring farmers’ due rewards is the key to maintaining the trusting relationship between farmers and governments during the rural tourism development.

Third, farmers’ perceived risk has a moderating effect between government trust and participation intention in the rural tourism. The perceived risk negatively moderates the relationship between government trust and farmers’ perceived usefulness of rural tourism. Specifically, at the same level of government trust, high perceived risks pose more negative effects on farmers’ perceived usefulness of rural tourism than low perceived risks. This maybe because the current development of rural tourism faces multiple influences from markets, resources, technology, and natural conditions, and the expected outcomes of farmers’ participation are uncertain. In the case of high perceived risk, even if the level of government trust is high, farmers still perceive rural tourism as impractical and tend to maintain the status quo and reduce the probability of participation. Thus, by introducing the perceived risk into the extended TAM model, this study can more reasonably explain the relationship between government trust and farmers’ participation in the rural tourism and the formation mechanism of farmers’ rural tourism participation. However, the moderating effect of farmers’ perceived risk on the relationship between government trust and perceived ease of use has not been found. This maybe that in the context of the “blossoming” development of rural tourism in China in recent years, farmers generally have certain knowledge about rural tourism, but due to the lack of participation and low level of participation, farmers tend to form the habitual perception that rural tourism is easy to participate in and ignore the conditions of participation, thus causing an insignificant moderating effect. In addition, the moderating effect of perceived risk on the relationship between government trust and farmers’ participation intention was not significant. The possible reason maybe that with a high level of government trust, farmers trust that government policies and practices can protect their livelihoods, thereby forming a “wait-and-see” mentality. As a result, their participation intentions will not be affected regardless of the level of perceived risk.

### Theoretical contribution

The TAM model is a theoretical model often used by domestic and international scholars to study technology adoption, consumer purchase, and farm participation behaviors. As the expansion of its explanation scope and the continuous exploration of scholars, the explanatory power of the model is enhanced with the addition of other variables. However, TAM models have not been studied in-depth in the field of tourism participation. In rural tourism research, farmers’ participation in tourism industry development and sharing the fruits of development have become the focus of rural tourism research in recent years (Wondirada and Ewnetub, 2019). However, few scholars have applied the TAM model to study farmers’ participation intention in the rural tourism. This study breaks through the previous research idea of mainly focusing on the impact of technology functionality on individual acceptance behavior, and extends the traditional TAM model by incorporating government trust variables. Meanwhile, the variable of perceived risk as a moderator is introduced to construct an analytical model of farmers’ participation intention in the rural tourism based on the extended TAM model.

This study points out the fundamental role of the TAM model in the study of farmers’ rural tourism participation behavior, and reveals that government trust has a non-negligible and important influence on farmers’ participation in the rural tourism. In addition, the role and mechanism of perceived risk in the relationship between government trust and farmers’ participation intention are examined through moderating effect analysis. An interesting finding is that government trust can significantly promote farmers’ rural tourism participation behavior. However, this effect is mainly mediated through perceived usefulness and perceived ease of use. We further explore the boundary conditions under which the mediating effect acts and find that the facilitation effect of government trust on farmers’ participation intention in the rural tourism is moderated by perceived risk. The level of farmers’ perceived risk in rural tourism participation behavior significantly moderates the relationship between government trust and perceived usefulness. The higher the perceived risk of farmers, the more difficulties in maintaining the positive influence relationship between government trust and perceived usefulness. However, the moderating effect of perceived risk on government trust and farmers’ perceived ease of use was not supported. This maybe because farmers perceive that rural tourism is easy to participate in and there is no need to consider the conditions of participation regardless of the level of perceived risk. Similarly, the moderating effect of perceived risk on the relationship between government trust and farmers’ participation intention was not supported. This suggests that with high levels of government trust, rural society’s “wait-and-see” negative ideology inhibits farmers’ participation intention. This study put forwards a new theoretical explanation for the current phenomena of “low participation rate and low participation level” and “high trust but low participation” in the rural tourism development, and provides some guiding suggestions to solve the problem of farmers’ participation behavior in the rural tourism development.

### Management implications

As China’s national strategy gradually tilts toward the countryside, the rural construction and development mode with rural tourism as the main selling point, has been widely replicated. However, the reality of lack of farmer participation in the rural tourism development is becoming increasingly evident. Therefore, combining the government trust and perceived risk in rural tourism development and based on the findings, this study proposes the following recommendations.

First, since farmers’ perceptions of the usefulness of participating in rural tourism positively influence their willingness, it is important to highlight the utility of rural tourism participation. With this purpose, firstly, the grassroots government should strengthen the high level of investment in local infrastructure and special facility of rural tourism. Specifically, integrate various agriculture-related funds to strengthen the connotation of rural tourism construction, and improve the quality of rural transportation, communications, medical care, education, and other public services, thereby creating a good development environment in the countryside, and enhancing the attractiveness of rural tourism destinations. Secondly, the grassroots government should promote the rural tourism publicity. To be specific, distribute illustrated and easy-to-understand industry knowledge booklets; perform face to face interview to promote the development of policy mechanisms, development prospects, well-being treatment, and etc.; utilize multi-steps to stimulate the main presence of farmers. In addition, the government can consciously select those farmers who have become rich in the rural tourism development as leaders and typical figures for rural advice to play the incentive demonstration effect among farmers. At the same time, organize some farmers to study outside to strengthen the cognition of usefulness of rural tourism. Thirdly, the grassroots government should combine with enterprises, Farmer Cooperative Organizations, and farmers to establish a stable interrelated benefit mechanism to provide farmers with economic income or employment opportunities in the form of dividend on shares. This can encourage employment and entrepreneurship for farmers, and attract the young people to return hometown to start their businesses and employment. Additionally, it can also guide and regulate farmers to carry out traditional handicraft processing and manufacturing, rural farm caravans, and local specialties sales. Based on this, farmers can effectively share the dividends of rural tourism development, enhance the sense of access, and resonate in the value sense.

Second, the perceived ease of use of rural tourism has a greater impact on farmers’ participation intention, so it is important to enhance the convenience of farmers’ intervention in rural tourism development. It is recommended to start from the following aspects: Firstly, optimize the external environment for farmers’ participation through organizational empowerment. The rural grassroots government should act as a leader to revitalize rural tourism economic cooperation organizations, take elements (land, forest land, housing, etc.), capital, technology, and labor into shares and other ways to attract farmers to join in; carry out cooperation and co-construction with the rural tourism industry in the form of economic organizations; integrate small farmer groups into an industrial development community with shared interests and risks; ensure farmers’ rights to information, participation, management, and supervision. In the meantime, it is necessary to enhance the ability of farmer households to cope with the market risk. Secondly, according to the genetic relationship, geographic-based relationship and industry relationship, the rural grassroots government should foster the development of various social organizations around the rural tourism industry featured by service, public welfare, or cooperation, such as rural tourism education association, rural tourism volunteer association, etc., to improve the convenience of participation. Thirdly, the rural grassroots government should give full play to the experts, scholars and Rural Sci-tech Special Commissioners, and organize scheduled or nonscheduled professional skills training according to the status of farmers’ professional skills and industrial development needs. Various vocational education and training methods, such as night school, new professional training courses, field observation, etc. should be utilized to continuously improve farmers’ familiarity with rural tourism, cultivate their awareness of participation, enhance their professional and technical ability and market awareness to adapt to the industrial development, and lower the quality threshold for participation.

Third, government trust significantly affects farmers’ rural tourism participation behavior, so increasing the trust in the government can reduce farmers’ concerns and greatly enhance their perceived usefulness and convenience of participation in the rural tourism. For this reason, the government should first ensure farmers share the fruits of tourism development through reasonable institutional arrangements and policy design, and provide reasonable compensation for farmers who bear loss in the tourism development (especially those not participating in the rural tourism), thereby keeping the continuous trust of farmers in the government. Secondly, the government should do a good job of top-level design, i.e., overall consider the rural land use, industrial development, habitat improvement, ecological protection, cultural heritage, etc.; implement the planning guidance; and promote the “integration of multiple regulations.” In the planning documents of industrial development, the articulated policy system should highlight the subjectivity of farmers. Relying on enterprises, government, cooperatives, farmers, and other organizational models, the innovative mechanism of rural tourism community participation encourages villagers and enterprises to participate in the rural tourism investment, operation, management, and services, especially the development and construction of projects with high participation and great benefits, which can prevent power and capital from controlling the rural tourism industry in general. Thirdly, the government should combine with relevant tourism enterprises to improve the infrastructure construction, protect rural resources and the beautiful environment in the process of development, improve public services, enhance the satisfaction of residents’ lives, and avoid negative impacts on the lives of rural residents. Fourthly, the government can provide assistance to farmers, care about their living conditions and development demands, simplify the related procedures, proactively reduce the obstacles for farmers to participate in the rural tourism, supply farmers with more opportunities to participate in the rural tourism through publicity, training, and collocation, etc. Fifthly, the government should timely upload and disseminate rural tourism-related policy documents and instructions, and combine with the actual grassroots organizations to carry out a variety of forms of learning and interpretation activities. With the help of the local government website, the official public WeChat, or the official microblog, policy documents can be timely and accurately obtained, and the information such as major matters and relevant project construction can be timely and objectively disclosed.

Fourth, the perceived risk has a positive impact on farmers’ participation intention in the rural tourism and government trust, so reducing farmers’ risk concerns can increase their optimism to participate in the rural tourism development. For this reason, we have the following recommendations: Firstly, the government should strengthen the interpretation and propagation of agriculture-related policies, and build a public interaction and mutual assistance platform led by village cadres and rural tourism operators through traditional means such as organizing the experiences exchange of rural tourism leaders and typical figures, supplemented by some new modes of rural socialization such as Internet learning (e.g., WeChat groups). This can better take the advantage of interpersonal dissemination of information in the acquaintance society and make it easier for farmers to access the information related to rural tourism development. Then, the market awareness and risk identification ability of farmers should be improved to reduce their fear of participating in the rural tourism. Secondly, the grassroots government, industrial operation companies, and farmer organizations should establish a coordination mechanism for distribution, delineate the minimum proportional share of annual profits in the income entitlements and labor income of farmers’ groups with dividends from land, forestry rights, and housing shares, supply farmers with reasonable protection in social security, medical insurance, and pension insurance by the law to ensure that farmers’ income will not be affected due to market fluctuations in the development of rural tourism industry, and provide stable and sustainable income treatment to reduce their risk aversion. Thirdly, given the benign role of traditional political participation in promoting government trust and reducing the risk perception, the government should strengthen farmers’ political participation, open up channels to reflect farmers’ social and public opinions, enhance the accessibility and feasibility of farmers’ democratic participation, and continuously broaden the breadth and depth of farmers’ participation in grassroots governance. Meanwhile, we should also establish the concept of active response, collect farmers’ complaints and opinions promptly, provide timely feedback and implement solutions, and properly deal with farmers’ urgent, difficult and worrying problems and give back as soon as possible.

### Limitations and future research directions

This study integrated the influence of variables such as the TAM model, government trust, and perceived risk on farmers’ participation intention in the rural tourism. Although complex antecedent variables and the formation mechanism of farmers’ rural tourism participation decisions have been considered, farmers’ government trust may still be influenced by other variables such as community attachment, which can be subsequently incorporated into the research model.

The literature research and focus group methods were employed in the study to select relevant measurement variables, which are inevitably subjective. The questionnaire design should be improved to more accurately incorporate different measurement aspects of the relevant variables into consideration in the future. For example, in the perceived usefulness index, elements such as “participation in management” and “environmental improvement” can be included to make the hypothesis test more convincing.

Although this study used the questionnaire method to collect cross-sectional data, respondents may still differ from typical farmers in terms of their willingness to participate in interviews, leisure time, familiarity with the researcher, and likelihood of using the Internet. At the same time, farmers’ trust in government is the result of a long-term interaction between farmers and governments, and it is necessary to conduct tracking studies to closely follow the dynamic change process of farmers’ trust in government. Therefore, future research can choose cross-time dimensional data to explore the longitudinal evolution pattern of farmers’ participation intention in the rural tourism, effectively avoiding the limitations of cross-sectional data.

## Data availability statement

The raw data supporting the conclusions of this article will be made available by the author, without undue reservation.

## Ethics statement

Ethical review and approval were not required for the study involving human participants in accordance with the local legislation and institutional requirements. Written informed consent to participate in this study was not required from the participants in accordance with the national legislation and institutional requirements.

## Author contributions

XY was mainly responsible for the work, including the conception and design of the study, performing the statistical analysis, organizing the database, and writing sections of the manuscript.

## Funding

This manuscript is supported by 2021 National Social Science Foundation “Temporal and spatial differentiation law and management response of tourism industry ecologization in China (21BGL148)”.

## Conflict of interest

The author declares that the research was conducted in the absence of any commercial or financial relationships that could be construed as a potential conflict of interest.

## Publisher’s note

All claims expressed in this article are solely those of the authors and do not necessarily represent those of their affiliated organizations, or those of the publisher, the editors and the reviewers. Any product that may be evaluated in this article, or claim that may be made by its manufacturer, is not guaranteed or endorsed by the publisher.
